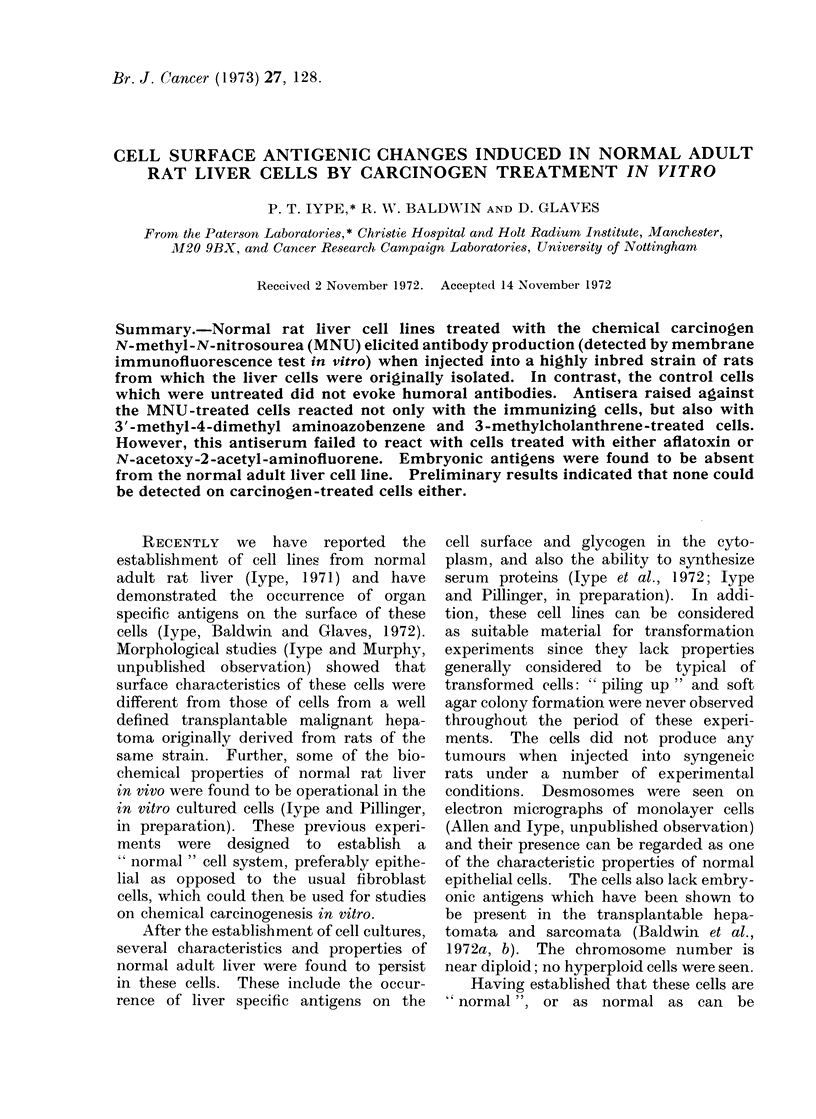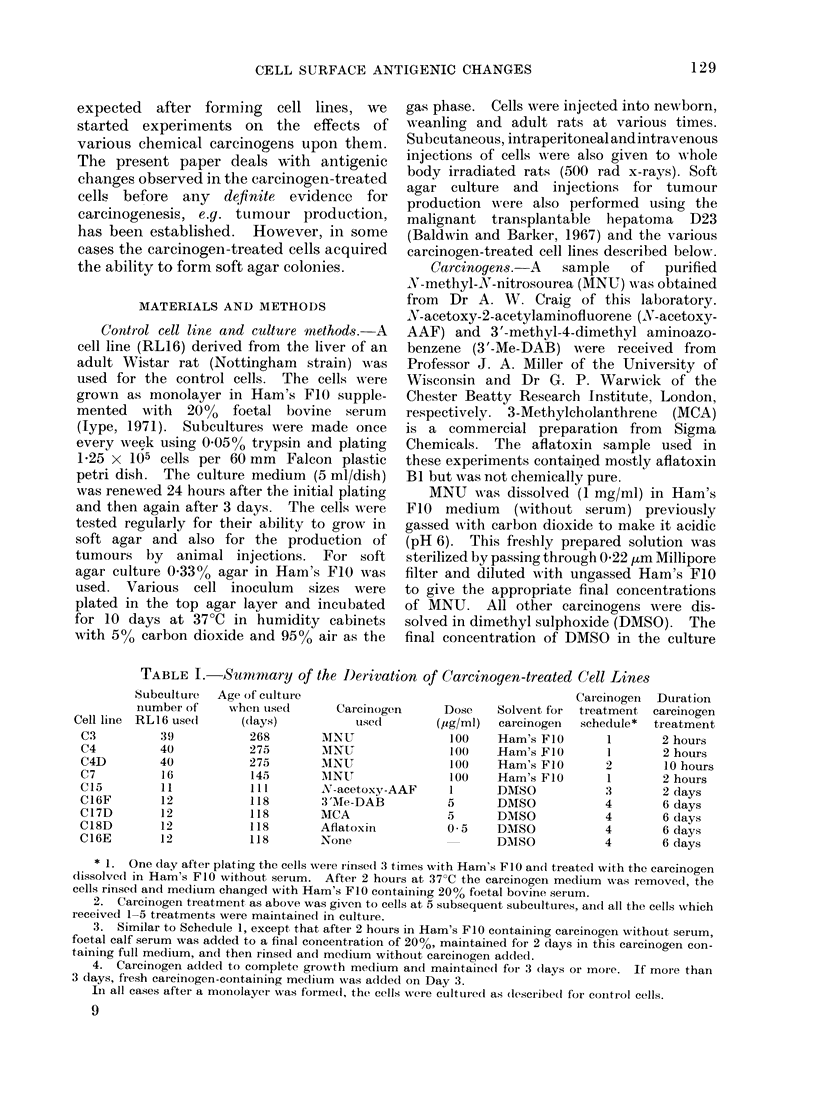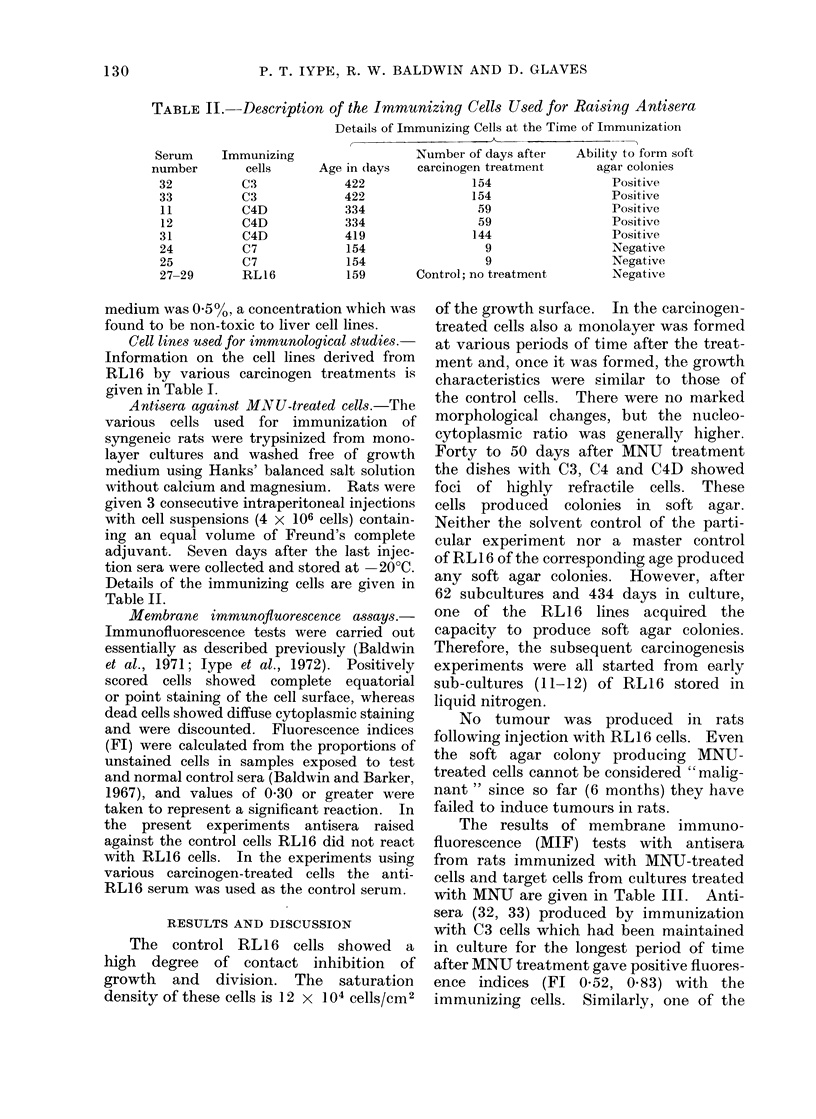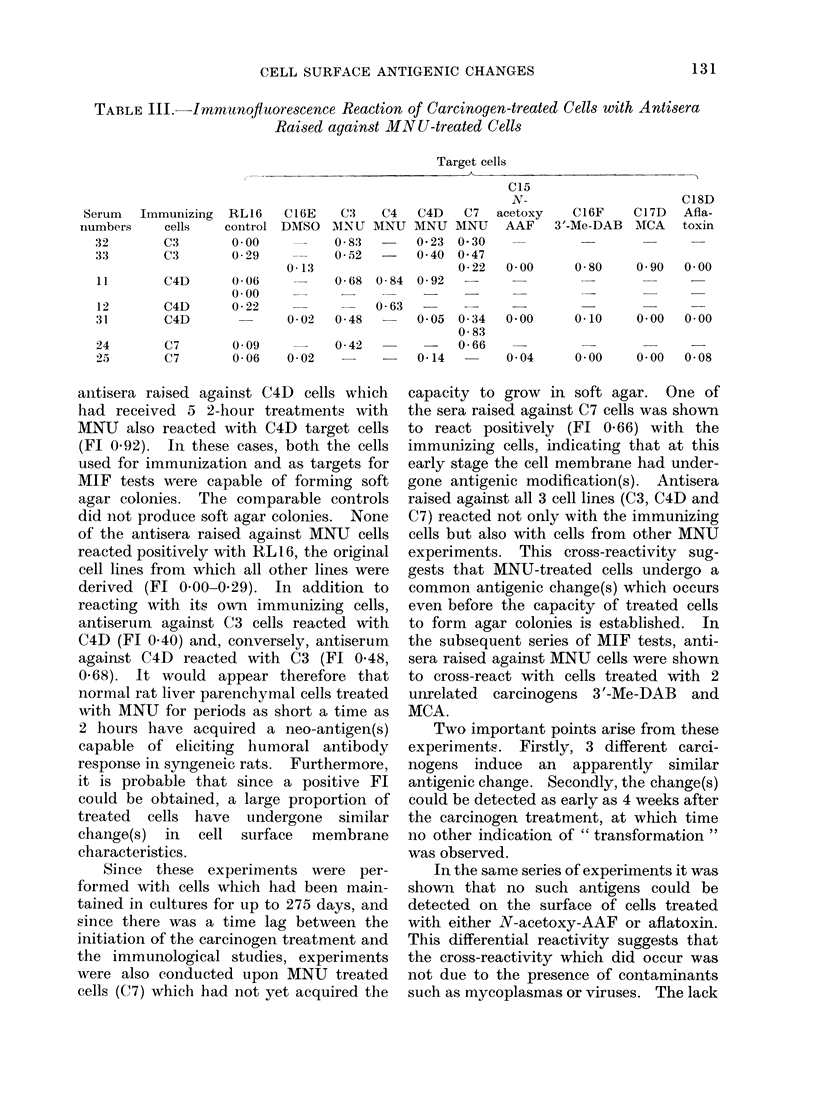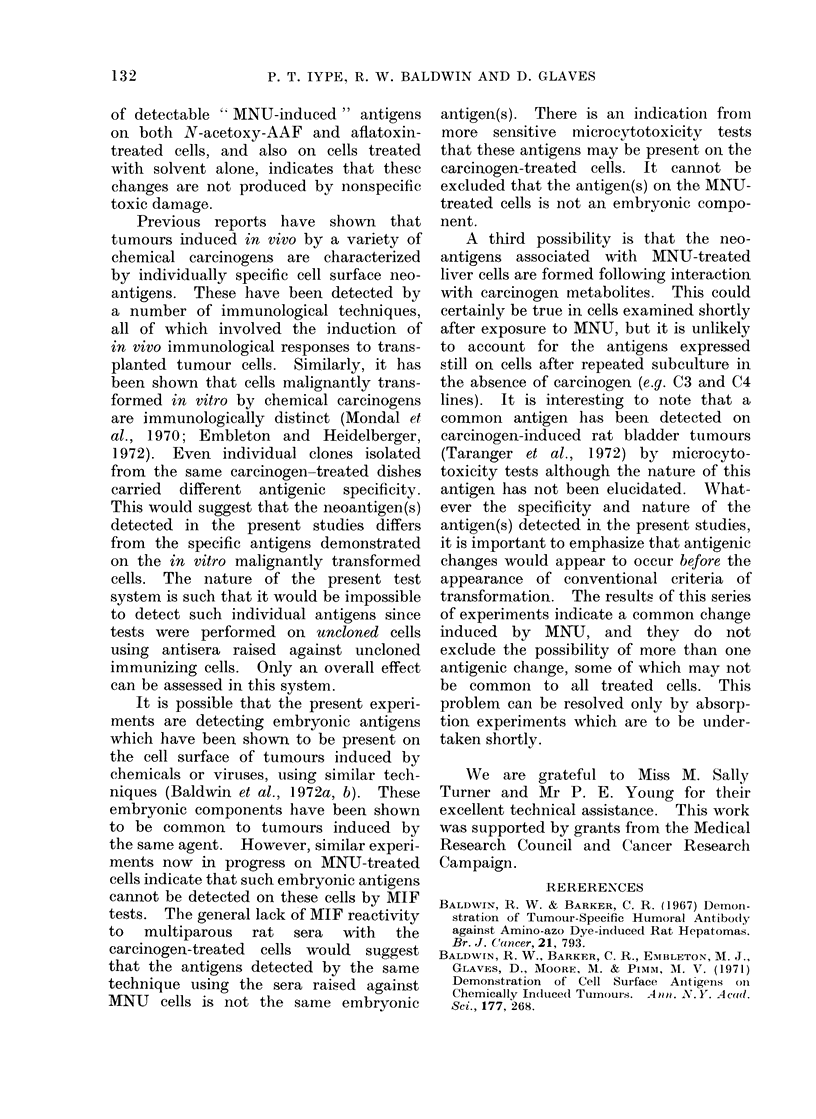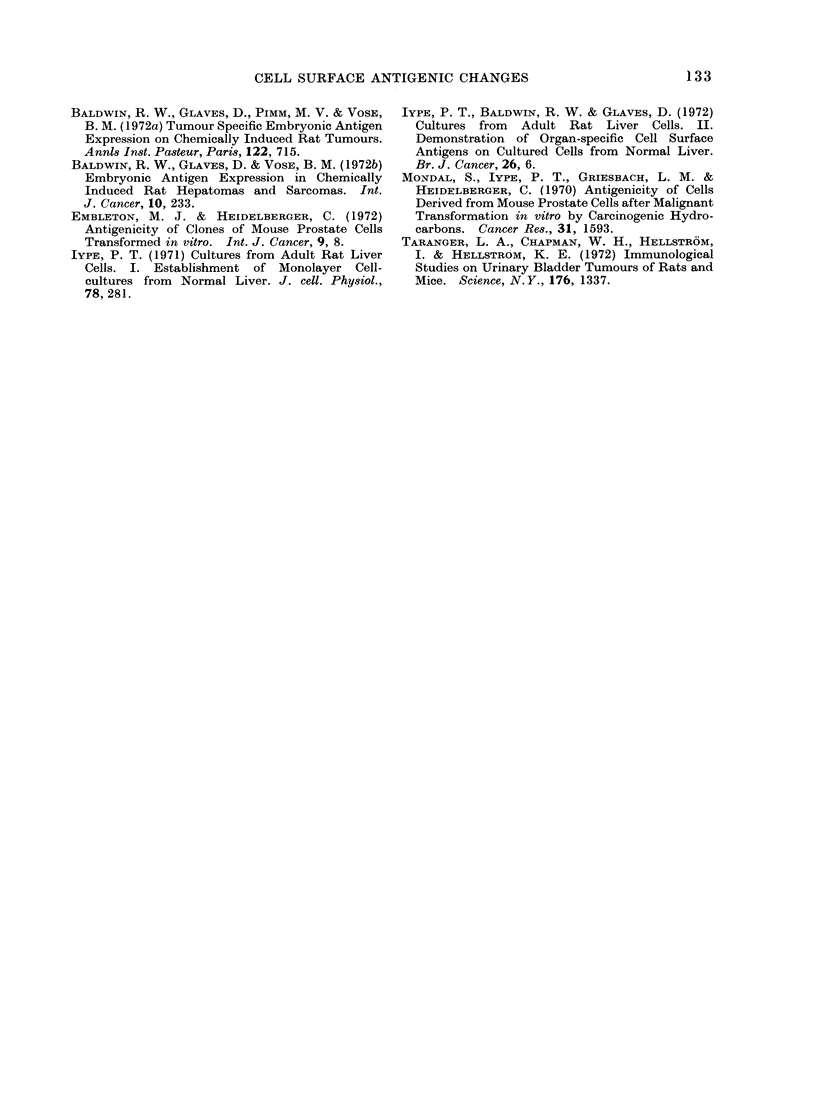# Cell Surface Antigenic Changes Induced in Normal Adult Rat Liver Cells by Carcinogen Treatment in vitro

**DOI:** 10.1038/bjc.1973.17

**Published:** 1973-02

**Authors:** P. T. Iype, R. W. Baldwin, D. Glaves

## Abstract

Normal rat liver cell lines treated with the chemical carcinogen *N*-methyl-*N*-nitrosourea (MNU) elicited antibody production (detected by membrane immunofluorescence test *in vitro*) when injected into a highly inbred strain of rats from which the liver cells were originally isolated. In contrast, the control cells which were untreated did not evoke humoral antibodies. Antisera raised against the MNU-treated cells reacted not only with the immunizing cells, but also with 3'-methyl-4-dimethyl aminoazobenzene and 3-methylcholanthrene-treated cells. However, this antiserum failed to react with cells treated with either aflatoxin or *N*-acetoxy-2-acetyl-aminofluorene. Embryonic antigens were found to be absent from the normal adult liver cell line. Preliminary results indicated that none could be detected on carcinogen-treated cells either.


Br. J. Cancer (1973) 27, 128.

### CELL SURFACE ANTIGENIC CHANGES INDUCED IN NORMAL ADULT RAT LIVER CELLS BY CARCINOGEN TREATMENT *IN VITRO*

P. T. IYPE,* R. W. BALDWIN AND D. GLAVES

*From the Paterson Laboratories,* Christie Hospital and Holt Radium Institute, Manchester, M20 9BX, and Cancer Research Campaign Laboratories, University of Nottingham*

Received 2 November 1972. Accepted 14 November 1972

**Summary.—Normal rat liver cell lines treated with the chemical carcinogen *N*-methyl-*N*-nitrosourea (MNU) elicited antibody production (detected by membrane immunofluorescence test *in vitro*) when injected into a highly inbred strain of rats from which the liver cells were originally isolated. In contrast, the control cells which were untreated did not evoke humoral antibodies. Antisera raised against the MNU-treated cells reacted not only with the immunizing cells, but also with 3′-methyl-4-dimethyl aminoazobenzene and 3-methylcholanthrene-treated cells. However, this antiserum failed to react with cells treated with either aflatoxin or *N*-acetoxy-2-acetyl-aminofluorene. Embryonic antigens were found to be absent from the normal adult liver cell line. Preliminary results indicated that none could be detected on carcinogen-treated cells either.**

RECENTLY we have reported the establishment of cell lines from normal adult rat liver (Iype, 1971) and have demonstrated the occurrence of organ specific antigens on the surface of these cells (Iype, Baldwin and Glaves, 1972). Morphological studies (Iype and Murphy, unpublished observation) showed that surface characteristics of these cells were different from those of cells from a well defined transplantable malignant hepatoma originally derived from rats of the same strain. Further, some of the biochemical properties of normal rat liver *in vivo* were found to be operational in the *in vitro* cultured cells (Iype and Pillinger, in preparation). These previous experiments were designed to establish a " normal " cell system, preferably epithelial as opposed to the usual fibroblast cells, which could then be used for studies on chemical carcinogenesis *in vitro*.

After the establishment of cell cultures, several characteristics and properties of normal adult liver were found to persist in these cells. These include the occurrence of liver specific antigens on the cell surface and glycogen in the cytoplasm, and also the ability to synthesize serum proteins (Iype *et al.*, 1972; Iype and Pillinger, in preparation). In addition, these cell lines can be considered as suitable material for transformation experiments since they lack properties generally considered to be typical of transformed cells: " piling up " and soft agar colony formation were never observed throughout the period of these experiments. The cells did not produce any tumours when injected into syngeneic rats under a number of experimental conditions. Desmosomes were seen on electron micrographs of monolayer cells (Allen and Iype, unpublished observation) and their presence can be regarded as one of the characteristic properties of normal epithelial cells. The cells also lack embryonic antigens which have been shown to be present in the transplantable hepatomata and sarcomata (Baldwin *et al.*, 1972*a*, *b*). The chromosome number is near diploid; no hyperploid cells were seen.

Having established that these cells are " normal ", or as normal as can be expected after forming cell lines, we started experiments on the effects of various chemical carcinogens upon them. The present paper deals with antigenic changes observed in the carcinogen-treated cells before any *definite* evidence for carcinogenesis, *e.g.* tumour production, has been established. However, in some cases the carcinogen-treated cells acquired the ability to form soft agar colonies.

### MATERIALS AND METHODS

*Control cell line and culture methods.*—A cell line (RL16) derived from the liver of an adult Wistar rat (Nottingham strain) was used for the control cells. The cells were grown as monolayer in Ham's F10 supplemented with 20% foetal bovine serum (Iype, 1971). Subcultures were made once every week using 0·05% trypsin and plating $1{\cdot}25 \times 10^5$ cells per 60 mm Falcon plastic petri dish. The culture medium (5 ml/dish) was renewed 24 hours after the initial plating and then again after 3 days. The cells were tested regularly for their ability to grow in soft agar and also for the production of tumours by animal injections. For soft agar culture 0·33% agar in Ham's F10 was used. Various cell inoculum sizes were plated in the top agar layer and incubated for 10 days at 37°C in humidity cabinets with 5% carbon dioxide and 95% air as the gas phase. Cells were injected into newborn, weanling and adult rats at various times. Subcutaneous, intraperitoneal and intravenous injections of cells were also given to whole body irradiated rats (500 rad x-rays). Soft agar culture and injections for tumour production were also performed using the malignant transplantable hepatoma D23 (Baldwin and Barker, 1967) and the various carcinogen-treated cell lines described below.

*Carcinogens.*—A sample of purified *N*-methyl-*N*-nitrosourea (MNU) was obtained from Dr A. W. Craig of this laboratory. *N*-acetoxy-2-acetylaminofluorene (*N*-acetoxy-AAF) and 3′-methyl-4-dimethyl aminoazobenzene (3′-Me-DAB) were received from Professor J. A. Miller of the University of Wisconsin and Dr G. P. Warwick of the Chester Beatty Research Institute, London, respectively. 3-Methylcholanthrene (MCA) is a commercial preparation from Sigma Chemicals. The aflatoxin sample used in these experiments contained mostly aflatoxin B1 but was not chemically pure.

MNU was dissolved (1 mg/ml) in Ham's F10 medium (without serum) previously gassed with carbon dioxide to make it acidic (pH 6). This freshly prepared solution was sterilized by passing through 0·22 μm Millipore filter and diluted with ungassed Ham's F10 to give the appropriate final concentrations of MNU. All other carcinogens were dissolved in dimethyl sulphoxide (DMSO). The final concentration of DMSO in the culture

TABLE I.—*Summary of the Derivation of Carcinogen-treated Cell Lines*

| Cell line | Subculture number of RL16 used | Age of culture when used (days) | Carcinogen used | Dose (μg/ml) | Solvent for carcinogen | Carcinogen treatment schedule* | Duration carcinogen treatment |
|---|---|---|---|---|---|---|---|
| C3 | 39 | 268 | MNU | 100 | Ham's F10 | 1 | 2 hours |
| C4 | 40 | 275 | MNU | 100 | Ham's F10 | 1 | 2 hours |
| C4D | 40 | 275 | MNU | 100 | Ham's F10 | 2 | 10 hours |
| C7 | 16 | 145 | MNU | 100 | Ham's F10 | 1 | 2 hours |
| C15 | 11 | 111 | *N*-acetoxy-AAF | 1 | DMSO | 3 | 2 days |
| C16F | 12 | 118 | 3′Me-DAB | 5 | DMSO | 4 | 6 days |
| C17D | 12 | 118 | MCA | 5 | DMSO | 4 | 6 days |
| C18D | 12 | 118 | Aflatoxin | 0·5 | DMSO | 4 | 6 days |
| C16E | 12 | 118 | None | — | DMSO | 4 | 6 days |

* 1. One day after plating the cells were rinsed 3 times with Ham's F10 and treated with the carcinogen dissolved in Ham's F10 without serum. After 2 hours at 37°C the carcinogen medium was removed, the cells rinsed and medium changed with Ham's F10 containing 20% foetal bovine serum.

2. Carcinogen treatment as above was given to cells at 5 subsequent subcultures, and all the cells which received 1–5 treatments were maintained in culture.

3. Similar to Schedule 1, except that after 2 hours in Ham's F10 containing carcinogen without serum, foetal calf serum was added to a final concentration of 20%, maintained for 2 days in this carcinogen containing full medium, and then rinsed and medium without carcinogen added.

4. Carcinogen added to complete growth medium and maintained for 3 days or more. If more than 3 days, fresh carcinogen-containing medium was added on Day 3.

In all cases after a monolayer was formed, the cells were cultured as described for control cells.

TABLE II.—*Description of the Immunizing Cells Used for Raising Antisera*

| Serum number | Immunizing cells | Details of Immunizing Cells at the Time of Immunization: Age in days | Number of days after carcinogen treatment | Ability to form soft agar colonies |
|---|---|---|---|---|
| 32 | C3 | 422 | 154 | Positive |
| 33 | C3 | 422 | 154 | Positive |
| 11 | C4D | 334 | 59 | Positive |
| 12 | C4D | 334 | 59 | Positive |
| 31 | C4D | 419 | 144 | Positive |
| 24 | C7 | 154 | 9 | Negative |
| 25 | C7 | 154 | 9 | Negative |
| 27–29 | RL16 | 159 | Control; no treatment | Negative |

medium was 0·5%, a concentration which was found to be non-toxic to liver cell lines.

*Cell lines used for immunological studies.*—Information on the cell lines derived from RL16 by various carcinogen treatments is given in Table I.

*Antisera against MNU-treated cells.*—The various cells used for immunization of syngeneic rats were trypsinized from monolayer cultures and washed free of growth medium using Hanks' balanced salt solution without calcium and magnesium. Rats were given 3 consecutive intraperitoneal injections with cell suspensions ($4 \times 10^6$ cells) containing an equal volume of Freund's complete adjuvant. Seven days after the last injection sera were collected and stored at −20°C. Details of the immunizing cells are given in Table II.

*Membrane immunofluorescence assays.*—Immunofluorescence tests were carried out essentially as described previously (Baldwin *et al.*, 1971; Iype *et al.*, 1972). Positively scored cells showed complete equatorial or point staining of the cell surface, whereas dead cells showed diffuse cytoplasmic staining and were discounted. Fluorescence indices (FI) were calculated from the proportions of unstained cells in samples exposed to test and normal control sera (Baldwin and Barker, 1967), and values of 0·30 or greater were taken to represent a significant reaction. In the present experiments antisera raised against the control cells RL16 did not react with RL16 cells. In the experiments using various carcinogen-treated cells the anti-RL16 serum was used as the control serum.

### RESULTS AND DISCUSSION

The control RL16 cells showed a high degree of contact inhibition of growth and division. The saturation density of these cells is $12 \times 10^4$ cells/cm$^2$ of the growth surface. In the carcinogen-treated cells also a monolayer was formed at various periods of time after the treatment and, once it was formed, the growth characteristics were similar to those of the control cells. There were no marked morphological changes, but the nucleo-cytoplasmic ratio was generally higher. Forty to 50 days after MNU treatment the dishes with C3, C4 and C4D showed foci of highly refractile cells. These cells produced colonies in soft agar. Neither the solvent control of the particular experiment nor a master control of RL16 of the corresponding age produced any soft agar colonies. However, after 62 subcultures and 434 days in culture, one of the RL16 lines acquired the capacity to produce soft agar colonies. Therefore, the subsequent carcinogenesis experiments were all started from early sub-cultures (11–12) of RL16 stored in liquid nitrogen.

No tumour was produced in rats following injection with RL16 cells. Even the soft agar colony producing MNU-treated cells cannot be considered "malignant" since so far (6 months) they have failed to induce tumours in rats.

The results of membrane immunofluorescence (MIF) tests with antisera from rats immunized with MNU-treated cells and target cells from cultures treated with MNU are given in Table III. Antisera (32, 33) produced by immunization with C3 cells which had been maintained in culture for the longest period of time after MNU treatment gave positive fluorescence indices (FI 0·52, 0·83) with the immunizing cells. Similarly, one of the

TABLE III.—*Immunofluorescence Reaction of Carcinogen-treated Cells with Antisera Raised against MNU-treated Cells*

| | | Target cells | | | | | | | | | |
|---|---|---|---|---|---|---|---|---|---|---|---|
| Serum numbers | Immunizing cells | RL16 control | C16E DMSO | C3 MNU | C4 MNU | C4D MNU | C7 MNU | C15 *N*-acetoxy AAF | C16F 3′-Me-DAB | C17D MCA | C18D Afla-toxin |
| 32 | C3 | 0·00 | — | 0·83 | — | 0·23 | 0·30 | — | — | — | — |
| 33 | C3 | 0·29 | — | 0·52 | — | 0·40 | 0·47 | | | | |
| | | | 0·13 | | | | 0·22 | 0·00 | 0·80 | 0·90 | 0·00 |
| 11 | C4D | 0·06 | — | 0·68 | 0·84 | 0·92 | — | — | — | — | — |
| | | 0·00 | — | — | — | — | — | — | — | — | — |
| 12 | C4D | 0·22 | — | — | 0·63 | — | — | — | — | — | — |
| 31 | C4D | — | 0·02 | 0·48 | — | 0·05 | 0·34 | 0·00 | 0·10 | 0·00 | 0·00 |
| | | | | | | | 0·83 | | | | |
| 24 | C7 | 0·09 | — | 0·42 | — | — | 0·66 | — | — | — | — |
| 25 | C7 | 0·06 | 0·02 | — | — | 0·14 | — | 0·04 | 0·00 | 0·00 | 0·08 |

antisera raised against C4D cells which had received 5 2-hour treatments with MNU also reacted with C4D target cells (FI 0·92). In these cases, both the cells used for immunization and as targets for MIF tests were capable of forming soft agar colonies. The comparable controls did not produce soft agar colonies. None of the antisera raised against MNU cells reacted positively with RL16, the original cell lines from which all other lines were derived (FI 0·00–0·29). In addition to reacting with its own immunizing cells, antiserum against C3 cells reacted with C4D (FI 0·40) and, conversely, antiserum against C4D reacted with C3 (FI 0·48, 0·68). It would appear therefore that normal rat liver parenchymal cells treated with MNU for periods as short a time as 2 hours have acquired a neo-antigen(s) capable of eliciting humoral antibody response in syngeneic rats. Furthermore, it is probable that since a positive FI could be obtained, a large proportion of treated cells have undergone similar change(s) in cell surface membrane characteristics.

Since these experiments were performed with cells which had been maintained in cultures for up to 275 days, and since there was a time lag between the initiation of the carcinogen treatment and the immunological studies, experiments were also conducted upon MNU treated cells (C7) which had not yet acquired the capacity to grow in soft agar. One of the sera raised against C7 cells was shown to react positively (FI 0·66) with the immunizing cells, indicating that at this early stage the cell membrane had undergone antigenic modification(s). Antisera raised against all 3 cell lines (C3, C4D and C7) reacted not only with the immunizing cells but also with cells from other MNU experiments. This cross-reactivity suggests that MNU-treated cells undergo a common antigenic change(s) which occurs even before the capacity of treated cells to form agar colonies is established. In the subsequent series of MIF tests, antisera raised against MNU cells were shown to cross-react with cells treated with 2 unrelated carcinogens 3′-Me-DAB and MCA.

Two important points arise from these experiments. Firstly, 3 different carcinogens induce an apparently similar antigenic change. Secondly, the change(s) could be detected as early as 4 weeks after the carcinogen treatment, at which time no other indication of " transformation " was observed.

In the same series of experiments it was shown that no such antigens could be detected on the surface of cells treated with either *N*-acetoxy-AAF or aflatoxin. This differential reactivity suggests that the cross-reactivity which did occur was not due to the presence of contaminants such as mycoplasmas or viruses. The lack of detectable "MNU-induced" antigens on both *N*-acetoxy-AAF and aflatoxin-treated cells, and also on cells treated with solvent alone, indicates that these changes are not produced by nonspecific toxic damage.

Previous reports have shown that tumours induced *in vivo* by a variety of chemical carcinogens are characterized by individually specific cell surface neo-antigens. These have been detected by a number of immunological techniques, all of which involved the induction of *in vivo* immunological responses to transplanted tumour cells. Similarly, it has been shown that cells malignantly transformed *in vitro* by chemical carcinogens are immunologically distinct (Mondal *et al.*, 1970; Embleton and Heidelberger, 1972). Even individual clones isolated from the same carcinogen-treated dishes carried different antigenic specificity. This would suggest that the neoantigen(s) detected in the present studies differs from the specific antigens demonstrated on the *in vitro* malignantly transformed cells. The nature of the present test system is such that it would be impossible to detect such individual antigens since tests were performed on *uncloned* cells using antisera raised against uncloned immunizing cells. Only an overall effect can be assessed in this system.

It is possible that the present experiments are detecting embryonic antigens which have been shown to be present on the cell surface of tumours induced by chemicals or viruses, using similar techniques (Baldwin *et al.*, 1972*a*, *b*). These embryonic components have been shown to be common to tumours induced by the same agent. However, similar experiments now in progress on MNU-treated cells indicate that such embryonic antigens cannot be detected on these cells by MIF tests. The general lack of MIF reactivity to multiparous rat sera with the carcinogen-treated cells would suggest that the antigens detected by the same technique using the sera raised against MNU cells is not the same embryonic antigen(s). There is an indication from more sensitive microcytotoxicity tests that these antigens may be present on the carcinogen-treated cells. It cannot be excluded that the antigen(s) on the MNU-treated cells is not an embryonic component.

A third possibility is that the neo-antigens associated with MNU-treated liver cells are formed following interaction with carcinogen metabolites. This could certainly be true in cells examined shortly after exposure to MNU, but it is unlikely to account for the antigens expressed still on cells after repeated subculture in the absence of carcinogen (*e.g.* C3 and C4 lines). It is interesting to note that a common antigen has been detected on carcinogen-induced rat bladder tumours (Taranger *et al.*, 1972) by microcytotoxicity tests although the nature of this antigen has not been elucidated. Whatever the specificity and nature of the antigen(s) detected in the present studies, it is important to emphasize that antigenic changes would appear to occur *before* the appearance of conventional criteria of transformation. The results of this series of experiments indicate a common change induced by MNU, and they do not exclude the possibility of more than one antigenic change, some of which may not be common to all treated cells. This problem can be resolved only by absorption experiments which are to be undertaken shortly.

We are grateful to Miss M. Sally Turner and Mr P. E. Young for their excellent technical assistance. This work was supported by grants from the Medical Research Council and Cancer Research Campaign.

### RERERENCES

BALDWIN, R. W. & BARKER, C. R. (1967) Demonstration of Tumour-Specific Humoral Antibody against Amino-azo Dye-induced Rat Hepatomas. *Br. J. Cancer*, **21**, 793.

BALDWIN, R. W., BARKER, C. R., EMBLETON, M. J., GLAVES, D., MOORE, M. & PIMM, M. V. (1971) Demonstration of Cell Surface Antigens on Chemically Induced Tumours. *Ann. N.Y. Acad. Sci.*, **177**, 268.

Baldwin, R. W., Glaves, D., Pimm, M. V. & Vose, B. M. (1972*a*) Tumour Specific Embryonic Antigen Expression on Chemically Induced Rat Tumours. *Annls Inst. Pasteur, Paris*, **122**, 715.

Baldwin, R. W., Glaves, D. & Vose, B. M. (1972*b*) Embryonic Antigen Expression in Chemically Induced Rat Hepatomas and Sarcomas. *Int. J. Cancer*, **10**, 233.

Embleton, M. J. & Heidelberger, C. (1972) Antigenicity of Clones of Mouse Prostate Cells Transformed *in vitro*. *Int. J. Cancer*, **9**, 8.

Iype, P. T. (1971) Cultures from Adult Rat Liver Cells. I. Establishment of Monolayer Cell-cultures from Normal Liver. *J. cell. Physiol.*, **78**, 281.

Iype, P. T., Baldwin, R. W. & Glaves, D. (1972) Cultures from Adult Rat Liver Cells. II. Demonstration of Organ-specific Cell Surface Antigens on Cultured Cells from Normal Liver. *Br. J. Cancer*, **26**, 6.

Mondal, S., Iype, P. T., Griesbach, L. M. & Heidelberger, C. (1970) Antigenicity of Cells Derived from Mouse Prostate Cells after Malignant Transformation *in vitro* by Carcinogenic Hydrocarbons. *Cancer Res.*, **31**, 1593.

Taranger, L. A., Chapman, W. H., Hellström, I. & Hellström, K. E. (1972) Immunological Studies on Urinary Bladder Tumours of Rats and Mice. *Science, N.Y.*, **176**, 1337.